# Pregabalin Prescription for Neuropathic Pain and Fibromyalgia: A Descriptive Study Using Administrative Database in Japan

**DOI:** 10.1155/2018/2786151

**Published:** 2018-06-05

**Authors:** Mikito Hirakata, Satomi Yoshida, Sachiko Tanaka-Mizuno, Aki Kuwauchi, Koji Kawakami

**Affiliations:** ^1^Department of Pharmacoepidemiology, Graduate School of Medicine and Public Health, Kyoto University, Yoshidakonoe-cho, Sakyo-ku, Kyoto 606-8501, Japan; ^2^Toxicology and Pharmacokinetics Laboratories, Pharmaceutical Research Laboratories, Toray Industries, Inc., 10-1, Tebiro 6-chome, Kamakura, Kanagawa 248-8555, Japan; ^3^Department of Medical Statistics, Shiga University of Medical Science, Seta Tsukinowa-cho, Otsu, Shiga 520-2192, Japan

## Abstract

**Objective:**

To assess dose, characteristics, and coprescribed analgesics in patients newly prescribed pregabalin for neuropathic pain and fibromyalgia in Japan.

**Methods:**

Based on the medical and prescription information present in the Medical Data Vision database, we analyzed the initial and maximum daily doses, prescription period, coprescribed analgesics, and neuropathic pain-related disorders of patients newly prescribed pregabalin between 01 July 2010 and 31 December 2013.

**Results:**

A total of 45,331 patients (mean age 66.8 years, 48.7% men) were newly prescribed pregabalin during this period. The mean initial and maximum daily doses were 97.3 mg and 127.8 mg, respectively, and decreased yearly. The duration of the prescription period was 111.9 (mean) and 53 (median) days, and the frequently coprescribed analgesics included NSAIDs, opioids, and Neurotropin®. About one half of the patients had spinal disorders.

**Conclusion:**

In Japan during the period examined, the number of newly prescribed pregabalin users increased, but the initial and maximum daily doses decreased yearly after pregabalin went on the market. The maximum daily dose in Japan was lower than those reported in the USA and Europe. These differences might be associated with patient age and physical status and with anxiety about possible adverse events.

## 1. Introduction

Neuropathic pain and fibromyalgia are intractable chronic pains. Neuropathic pain is defined as “pain caused by a lesion or disease of the somatosensory system” and is classified as peripheral or central neuropathic pain according to the site of the lesion or disease [1, 2]. The prevalence of neuropathic pain was estimated at 6.9% to 10% in some countries [3]. Fibromyalgia is a disorder characterized by systemic pain accompanied by neuropsychiatric symptoms such as insomnia and depression and autonomic symptoms such as irritable bowel syndrome, gastroesophageal reflux disease, and over active bladder [4–6]. In Japan, the prevalence was estimated to be 1.7%–2.1% and about 60%–80% of sufferers were women [7, 8].

Pregabalin is a ligand for the *α*_2_ *δ* subunit of the calcium channel and is used world wide to treat seizure, generalized anxiety disorder, neuropathic pain, and fibromyalgia. Pregabalin is a first- and/or second-line recommendation for neuropathic pain in many guidelines [9–12] and was approved for fibromyalgia in the USA but not in Europe. In Japan, pregabalin was approved for postherpetic neuralgia in April, 2010, and current indications have been expanded to neuropathic pain and first-line recommendation in the guideline for pharmacologic treatment of neuropathic pain [13]. Pregabalin was also approved for fibromyalgia in June, 2012, in Japan.

Pregabalin is approved to be started at 150 mg daily and titrated up to a maintenance daily dose range. In Japan and the USA, this dose range is 300–600 mg for neuropathic pain and 300–450 mg for fibromyalgia, and in Europe, 150–600 mg for neuropathic pain. However, the efficacy of 150 mg daily was inconsistent [14–16]. In the USA or European observation studies, the mean maximum (or average) daily dose was less than 300 mg (lower limit of approved maintenance dose range in the USA and Japan) or many patients were prescribed <300 mg for neuropathic pain [17–22] and fibromyalgia [23–25].

An interim report of postmarketing surveillance for peripheral neuropathic pain in Japan [26] showed that the mean initial and maximum daily doses of pregabalin were less than the approved initial and maintenance doses. However, the patient population of this study was small (2010 patients), and the observation period was short (13 weeks). Thus, in the present study, the real-world pregabalin prescription for neuropathic pain and fibromyalgia in Japan was examined using the Medical Data Vision database, a large medical and prescription database.

## 2. Methods

### 2.1. Data Sources

This descriptive study was conducted using the data collected and aggregated by the Medical Data Vision (MDV) Co. Ltd. from the hospitals using a novel medical reimbursement system for hospitalization, the Diagnosis Procedure Combination/Per-Diem Payment System (DPC/PDPS) in Japan [27, 28]. In April 2016, 1667 hospitals had introduced the DPC/PDPS, encompassing a total of about 495,227 beds. These constituted about 20% of all hospitals and 55% of the total hospital beds in Japan [29]. In May 2016, this database contained the anonymized data of 14,390,000 patients from 247 hospitals. These data contain medical and prescription data from both inpatients and outpatients. Prescription data consisted of individual records each containing one set of information comprising drug name, content, prescription date, daily volume or number of drug formulation, and the number of days prescribed. The study protocol was approved by Kyoto University Graduate School and Faculty of Medicine, Ethics Committee (Kyoto, Japan, Application number E2507).

### 2.2. Patients

We selected patients newly prescribed pregabalin between 01 July 2010 and 31 December 2013. *Newly prescribed* was defined as a first prescription in the database with no prescribed pregabalin in the previous 90 days. The date of newly prescribed pregabalin was designated as the *first prescription date*. Patients were excluded when the hospital's data collection had started within 90 days before the first prescription date. Records of prescribed pregabalin “as-needed” were excluded and patients whose first prescription was only “as-needed” were excluded.

### 2.3. Daily Dose and Prescription Period

The *daily dose of pregabalin* was calculated from the content and the daily number of capsules. When there were several records on the same prescription date, daily dose and the number of the days prescribed were estimated based on the number of days until the next pregabalin prescription date. The *last prescription date* was defined as the last date of pregabalin prescription in the database or as the first date with no pregabalin prescription after 30 days plus the number of days of the last prescription. The *prescription period* was set to be the time between the first prescription date and the end of the time prescribed by the last prescription. When the duration of the prescription period was over 365 days, this duration was set at 365 days.

### 2.4. Coprescribed Drugs

*Coprescriptions* were defined as drugs that were coprescribed at the first prescription date of pregabalin, or before this first date but overlapping it and represcribed within 90 days after it. The number of kinds of coprescribed oral drugs was based on substance names. Coprescribed analgesic drugs were categorized as (1) first- and second-line drugs of the Japanese guideline (1-2-line drugs; oral formulation), including tricyclic antidepressants (TCAs; amitriptyline hydrochloride, imipramine hydrochloride, and nortriptyline hydrochloride), gabapentin, extract of cutaneous tissue of rabbit inoculated with vaccinia virus (Neurotropin), duloxetine hydrochloride, and mexiletine hydrochloride; (2) opioids, including fentanyl (transdermal patch), oxycodone (oral formulation), morphine (oral and injection formulations), buprenorphine (transdermal patch and oral mucosa patch), tramadol (oral and injection formulations), and acetaminophen/tramadol combination (oral formula); and (3) nonsteroidal anti-inflammatory drugs (NSAIDs; ATCcode M01A and N02B0, excluding pentazocine and acetaminophen/tramadol combination; oral formulation). Opioids were subcategorized by strength (weak (tramadol and acetaminophen/tramadol combination) and strong (the others)) and route (oral and nonoral).

### 2.5. Neuropathic Pain-Related Disorders

*Neuropathic pain-related disorders* were classified as follows: (1) spinal disorders (ICD-10 code: M47, M48, M50, M51, or M53), (2) postherpetic neuropathy (ICD-10 code: B02.2), (3) diabetic neuropathy (ICD-10 code: E10.4, E11.4, or E14.4), (4) cancer-related pain (disease name: cancer pain, or ICD-10 code: C00-C97 in combination with ICD-10 code: R52.1, R52.2, or R52.9), (5) trigeminal neuralgia (ICD-10 code: G50.0), (6) entrapment peripheral neuropathy of the upper limb (disease name: carpal-tunnel syndrome, Gion tunnel syndrome, cubital tunnel syndrome, or thoracic outlet syndrome), (7) other neuropathic pain (ICD-10 code: G62.9, G64, G96.9, or G98, not classified in the above categories), (8) fibromyalgia (disease name: fibromyalgia), and (9) others (not classified in the above categories).

### 2.6. Analgesic Drugs Prescribed after Pregabalin Discontinuation Period

Patients were analyzed whose pregabalin prescription period duration was less than 365 days. The *after pregabalin discontinuation period* was defined as the time between the day after the last prescription date and 90 days after the end of the pregabalin prescription period. The prescriptions of the analgesic drugs described above in this period were then summarized. The prescriptions after the pregabalin discontinuation period were compared with those during the pregabalin prescription period and categorized as follows: (1) *continued use*: when the same drug had been prescribed during the pregabalin prescription period without prescriptions of other drugs of the same category, (2) *new use*: when a drug or other drugs of the same category had not been prescribed during the pregabalin prescription period, (3) *changed/added drugs*: the drugs prescribed after the pregabalin discontinuation period were different from or added to other drugs of the same category prescribed during the pregabalin prescription period, (4) *changed/additions of the route*: the routes of the drugs coprescribed during the pregabalin prescription period were changed or new administration routes for the same drugs were added after the pregabalin discontinuation period, and (5) *changed/additions of the strength*: different strengths of opioids were prescribed after the pregabalin discontinuation period to change from or add to opioids prescribed during the pregabalin prescription period.

### 2.7. Statistical Analysis

Numerical data are presented as means ± their standard deviation, or as medians and their 25th and 75th percentiles, and categorical data are presented as numbers and percentages. Statistical comparisons were made using the *t*-test for numerical data and the chi-square test for categorical data and were conducted using the SAS version 9.4 (SAS Institute Inc.) statistical package.

## 3. Results

### 3.1. Newly Prescribed Pregabalin Patients

**Figure 1:**
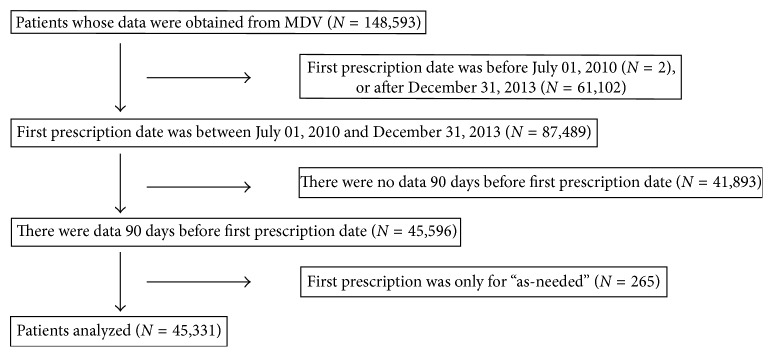
Patient selection flowchart.

**Figure 2:**
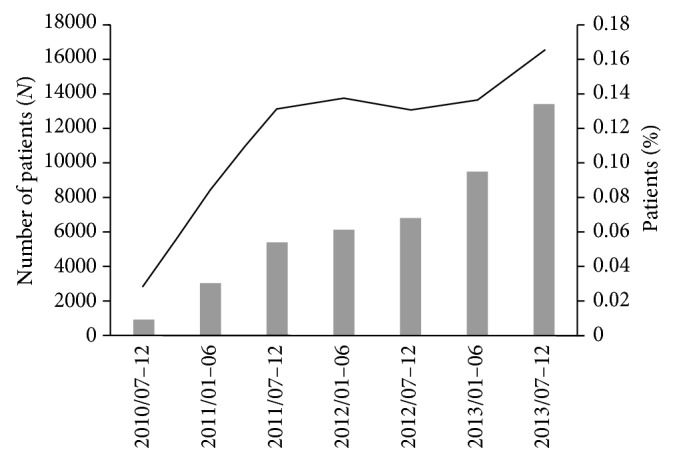
The number of patients newly prescribed pregabalin. Solid columns represent the number of patients newly prescribed pregabalin in each half year. Solid line represents the number of patients newly prescribed pregabalin in each half year divided by the total number of monthly patients in each half year in this database.

We obtained the data of 148,593 patients from the MDV database who had been prescribed pregabalin; 45,331 of those patients met the necessary criteria, and their data were analyzed further (Figure 1). The number of patients newly prescribed pregabalin increased dramatically in the first year after the launch of pregabalin in the second half of 2010. After that period, the number of these patients increased gradually (Figure 2).

### 3.2. Characteristics of Pregabalin-Prescribed Patients

**Figure 3:**
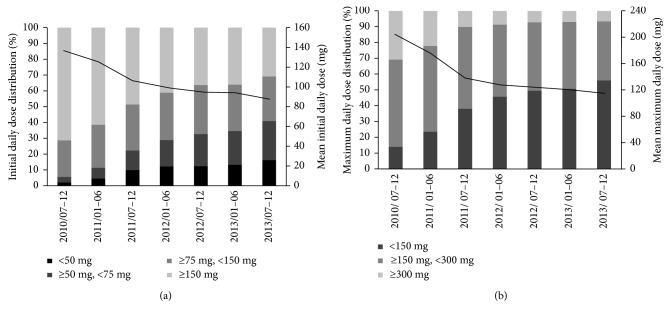
The initial (a) and maximum (b) daily doses. Solid columns represent the daily dose distribution. Solid line represents the mean daily dose.

Table 1 shows the characteristics of pregabalin-prescribed patients. At their first prescription date, 37,045 of the patients (81.7%) were prescribed pregabalin in an outpatient setting. The male ratio was 48.7%, and the mean age was 66.8 ± 13.9 years. The male ratio of inpatients was over 50%, and the mean age of inpatients was a little higher than that of outpatients. The mean initial daily dose and maximum daily dose were 97.3 ± 56.7 mg and 127.8 ± 87.0 mg, respectively, but these tended to decrease as time proceeded after the initial launch (Figure 3).

The mean and median durations of the prescription period were 111.9 ± 124.2 and 53 days, respectively. The duration of the prescription period was longer for outpatients than that for inpatients. The fraction of patients who changed from the initial daily dose to at least one another dose during the prescription period (change in dose) was only 26.9%, and these changes were higher for inpatients than they were for outpatients. The mean number of coprescribed oral drugs was 4.2 ± 3.7, and the number for inpatients was higher than that for outpatients.

The most frequently coprescribed analgesics for outpatients at the first pregabalin prescription date were NSAIDs (36.9%); the second most frequent was Neurotropin. Opioids were coprescribed for 5.7% of the outpatients, and most of these were oral formulas. For inpatients, the most frequently coprescribed analgesics were NSAIDs, followed by opioids; strong opioids were more frequently coprescribed than weak ones.

About one half of the outpatients had spinal disorders; other disorders, in descending order of frequency, included postherpetic neuropathy, cancer-related pain, and diabetic neuropathy. About 30% of inpatients had spinal disorders and about 20% had cancer-related pain.

To exclude the effects of short-trial pregabalin use on the results, a subgroup analysis of 12-month continuous users was conducted (Supplementary Table S1). Compared with the results of the main analysis, the mean maximum daily dose increased to 153.5 ± 106.2, 16.4% of the patients were prescribed ≥300 mg as the maximum dose, and 51.7% of them underwent a change in dose. Other parameters were comparable to those in the main analysis.

### 3.3. Pregabalin Prescription Patterns according to Gender and Age

Table 2 shows the pregabalin prescription patterns according to gender and age. The initial and maximum daily doses prescribed for males were higher than those prescribed for females, and were also higher for patients younger than 65 years than for those 65 years or older. On the other hand, there were only small differences in the durations of the prescription period between males and females, while the duration of the prescription period for patients younger than 65 was shorter than that of those 65 years or older. The maximum daily dose tended to increase with increasing first daily doses. The prescription period tended to be shorter for those receiving initial daily doses of more than 75 mg/day.

### 3.4. Subgroup Analysis of Individual Disorders

The use of pregabalin and coprescribed drugs was analyzed in subgroups of the following individual disorders: spinal disorders, postherpetic neuropathy, diabetic neuropathy, cancer-related pain, trigeminal neuralgia, and entrapment peripheral neuropathy of the upper limb (Table 3). The male ratio was higher for diabetic neuropathy and cancer-related pain. The mean age of patients was greater than 60 years for all disorders. The initial and maximum daily doses of pregabalin tended to be slightly lower in outpatients with spinal disorders, diabetic neuropathy, and entrapment neuropathy of the upper limb than in those with other disorders. The maximum daily dose of pregabalin was higher in inpatients than in outpatients with spinal disorders, postherpetic neuropathy, cancer-related pain, and entrapment neuropathy of the upper limb. Inpatients tended to have shorter durations of the prescription period than did outpatients with spinal disorders, diabetic neuropathy, cancer-related pain, and trigeminal neuralgia.

Only about 5% of patients with cancer-related pain were coprescribed 1-2-line analgesic drugs, while these were prescribed for 10.4% to 16.1% of the patients with other disorders. By contrast, about half of the patients with cancer-related pain were coprescribed opioids, while at most 15% of the patients with other pain disorders received opioids. More inpatients were coprescribed opioids along with the first prescription than were outpatients. Smaller percentages of diabetic neuropathy patients (both inpatients and outpatients) as well as outpatients with trigeminal neuropathy and entrapment neuropathy of the upper limb were coprescribed NSAIDs.

### 3.5. After the Pregabalin Discontinuation Period

After the pregabalin discontinuation period (Table 4), 10.2% of patients were represcribed pregabalin, and 8.2% and 15.2% of patients were prescribed 1-2-line drugs and opioids, respectively. About 5% of patients continued receiving prescriptions of the same 1-2-line drugs, and 3.4% received new 1-2-line prescriptions, while 4.0% continued previously prescribed opioids, and 7.4% received new opioid prescriptions.

When individual pain disorders were analyzed separately (Supplementary Table S2), pregabalin was represcribed for less than 10% of postherpetic neuralgia and cancer-related pain patients. The frequency of prescribed opioids for cancer-related pain (50.2%) was highest in patients with these disorders, and most were continued prescriptions or changes/additions of the drugs/routes (all greater than 10%).

## 4. Discussion

The data for the 45,331 patients newly prescribed pregabalin included in the MDV database, showed that the number of new users increased considerably from the launch of pregabalin in the second half of 2010 to the second half of 2011, when this increase leveled off. Indications for pregabalin were expanded in 2012 and 2013; however, these expansions were for fibromyalgia and central neuropathic pain and had little affect on the number of pregabalin prescriptions, as there are fewer of these patients than those with the prior indications for peripheral neuropathic pain.

The initial daily dose was lower than the approved initial daily dose in Japan (150 mg), and decreased yearly; 60.8% of patients were prescribed less than the approved dose. The reason for this decrease may be due to the many adverse events observed early in the launch of pregabalin [16]. A notice for elderly patients was released by the Pharmaceuticals and Medical Devices Agency of Japan in July 2012 [30] stating that “In elderly patients, some cases of falls due to dizziness, somnolence, loss of consciousness, etc. leading to fractures have been reported; therefore extra caution should be exercised.” The initial daily doses prescribed for males were higher than those for females and were also higher for patients younger than 65 years than for those 65 years or older. Differences in physical status (weight and height) or excretory function and older age being a risk factor for early onset of adverse events, such as somnolence and dizziness [31], were considered possible reasons for these differences. The duration of the prescription period was shorter in those patients who received more than 75 mg/day as an initial daily dose. The incidence of adverse events in those receiving more than 75 mg/day may have increased, resulting in a shorter duration of the prescription period.

The maximum daily dose was lower than both the approved initial (150 mg) and maintenance (≥300 mg) daily doses in Japan; the proportions of patients prescribed less than approved doses were 47.4% and 91.0%, respectively. In the subgroup analysis of 12-month continuous users, the maximum daily dose (153.5 mg) was comparable to the approved initial daily dose in Japan, and the proportions of patients prescribed less than the approved doses were 38.6% and 83.6%, respectively. Such low doses were also reported in another Japanese study (134.9 mg) [16]. In USA and European studies, the maximum or average daily doses (about 170–280 mg) [18–24] were less than 300 mg and although not many patients were prescribed more than 300 mg (about 20–30%) [18, 19, 21, 23, 25], these numbers were still higher than those in Japan. In randomized control trials, the efficacy of 150 mg/day was inconsistent [14–16]. Especially in those conducted in Japan [32–34], only one study for postherpetic neuralgia had a 150 mg group as the maintenance dose, but significant effects were not shown [32]. The maximum daily dose in Japan was considered much lower, this might be associated with differences in age; the patients in this study (67 years old) were older than those in other studies (45–67 years old). There were also differences in physical status; Japanese people are generally less heavy and shorter than Westerners. Prescribing doctors may also have had anxiety about the reported adverse events; the initial daily dose was being decreased and many patients were prescribed the same daily dose during the prescription period.

The ratio of males, initial daily dose, maximum daily dose, and number of coprescribed opioids were higher in inpatients than in outpatients. The high ratio of males was considered to result from the high ratio of cancer-related pain patients and a low ratio of spinal disorders because there was a higher ratio of cancer-related pain in males and a lower ratio of males with spinal disorders. The higher ratio of coprescribed opioids in inpatients was observed for all disorders, including cancer-related pain. This may be because severer pain patients may more often be inpatients than outpatients.

The 1-2-line drugs were coprescribed for about 15% patients with all disorders except cancer-related pain, and NSAIDs were coprescribed for 20% to 40% of patients. The high frequency of coprescribed NSAIDs may be due to many patients also having nociceptive pain, another component of pain. Approximately half of the patients with cancer-related pain were coprescribed opioids, and one half of the patients were coprescribed NSAIDs. NSAIDs and opioids were recommended for cancer pain, and pregabalin was coprescribed for adjuvant analgesics or relief of neuropathic pain from cancer or cancer treatment and/or the coexistence of these pains.

This study had several strengths. First, a large population of patients who were prescribed pregabalin was analyzed about 10 times that of other drug-use investigations in Japan [15], and the maximum follow-up period was 4 times longer. Second, the database contained the data of patients who visited hospitals regardless of their kind of insurance. There were patients of a variety of ages, including later-stage elderly (75 or older) and a variety of jobs. Third, pregabalin was only approved in Japan for pain disorder, neuropathic pain, and fibromyalgia, and unapproved for other symptoms, such as seizure and generalized anxiety disorder; therefore, the analysis in this study was able to specialize in its use for pain.

There were also some limitations. First, the follow-up of patients was limited because this was a database of hospital-based data. After discontinuation of pregabalin, other analgesics were prescribed for about 30% of patients. When there were no data of analgesic prescriptions, it was not possible to know whether no analgesics had been prescribed or the patients had consulted a different hospital. However, these patients were considered to be unsatisfied with the effectiveness of pregabalin. Second, the reasons for discontinuation were not confirmed because the effects and the most adverse drug reactions of pregabalin, such as dizziness and somnolence, were not collected for this database. Third, there is a possibility of bias in the patient population because many small scale hospitals have not introduced the DPC/PDPS [29]. Moreover, as the DPC/PDPS is a system for hospitalization, this database has no data from hospitals without inpatient facilities and little data from small scale hospitals. Forth, the disorders for which pregabalin was prescribed were not confirmed. In this database, prescribed drugs and the disorders for which they were prescribed were not combined; therefore, for example, the neuropathic pain-related disorders were categorized from those treated in the same month of the first prescription date.

## 5. Conclusion

In Japan, the number of patients being newly prescribed pregabalin increased over the course of the study period, but the initial and maximum daily doses decreased yearly after pregabalin went on the market. The maximum daily dose in Japan was lower than those reported in the USA and Europe, which may be associated with differences in age and physical status and anxiety about possible adverse events.

## Figures and Tables

**Table 1:** Characteristics of pregabalin-prescribed patients.

	All	Outpatient	Inpatient	*P* value
*N*	45331	37045	8286	

Sex (male), *N* (%)	22085 (48.7)	17692 (47.8)	4393 (53.0)	<0.001

Age, mean (SD)	66.8 (13.9)	66.4 (14.0)	69.0 (13.3)	<0.001

Initial daily dose, mg				
Mean (SD)	97.3 (56.7)	95.5 (55.7)	105.4 (60.5)	<0.001

*N* (%)				
<50 mg	5737 (12.7)	4733 (12.8)	1004 (12.1)	—
≥50 mg, <75 mg	8691 (19.2)	7596 (20.5)	1095 (13.2)	—
≥75 mg, <150 mg	13139 (29.0)	10687 (28.8)	2452 (29.6)	—
≥150 mg	17764 (39.2)	14029 (37.9)	3735 (45.1)	—

Maximum daily dose, mg				
Mean (SD)	127.8 (87.0)	123.6 (82.5)	146.2 (102.5)	<0.001

*N* (%)				
<150 mg	21468 (47.4)	18162 (49.0)	3306 (39.9)	—
≥150 mg, <300 mg	19763 (43.6)	15913 (43.0)	3850 (46.5)	—
≥300 mg	4100 (9.0)	2970 (8.0)	1130 (13.6)	—

Prescription period, day				
Mean (SD)	111.9 (124.2)	118.5 (126.8)	82.6 (107.0)	<0.001
Median (25, 75 percentile)	53 (21, 163)	56 (21, 182)	36 (14, 94)	<0.001
Prescription period over 90 days, *N* (%)	16762 (37.0)	14595 (39.4)	2167 (26.2)	<0.001

Change in dose, *N*(%)	12205 (26.9)	9584 (25.9)	2621 (31.6)	<0.001

Coprescribed oral drugs, mean (SD)	4.2 (3.7)	3.9 (3.6)	5.7 (4.0)	<0.001

Coprescribed analgesics, *N* (%)				
1-2-Line drugs	4698 (10.4)	4017 (10.8)	681 (8.2)	<0.001
TCAs	615 (1.4)	473 (1.3)	142 (1.7)	—
Gabapentin	160 (0.4)	120 (0.3)	40 (0.5)	—
Neurotropin	3656 (8.1)	3256 (8.8)	400 (4.8)	—
Duloxetine	258 (0.6)	178 (0.5)	80 (1.0)	—
Mexiletine	217 (0.5)	162 (0.4)	55 (0.7)	—
Opioids	3843 (8.5)	2117 (5.7)	1726 (20.8)	<0.001
Oral opioids	3071 (6.8)	1825 (4.9)	1246 (15.0)	—
Nonoral opioids	790 (1.7)	275 (0.7)	515 (6.2)	—
Strong opioids	2219 (4.9)	954 (2.6)	1265 (15.3)	—
Weak opioids	1688 (3.7)	1184 (3.2)	504 (6.1)	—
NSAIDs	17303 (38.2)	13657 (36.9)	3646 (44.0)	<0.001

Neuropathic pain-related disorders^*∗*^, *N* (%)				
Spinal disorders	23502 (51.8)	21144 (57.1)	2358 (28.5)	<0.001
Postherpetic neuropathy	2795 (6.2)	2435 (6.6)	360 (4.3)	<0.001
Diabetic neuropathy	1478 (3.3)	1244 (3.4)	234 (2.8)	0.014
Cancer-related pain	3933 (8.7)	2172 (5.9)	1761 (21.3)	<0.001
Trigeminal neuralgia	780 (1.7)	713 (1.9)	67 (0.8)	<0.001
Entrapment peripheral neuropathy of the upper limb	1329 (2.9)	1251 (3.4)	78 (0.9)	<0.001
Other neuropathic pain disorders	11564 (25.5)	9728 (26.3)	1836 (22.2)	—
Fibromyalgia	153 (0.3)	127 (0.3)	26 (0.3)	0.681
Others	3178 (7.0)	1113 (3.0)	2065 (24.9)	—

1-2-line drugs: first- and second-line drugs of the Japanese guideline; TCAs: tricyclic antidepressants (amitriptyline hydrochloride, imipramine hydrochloride, and nortriptyline hydrochloride); neurotropin; extract of cutaneous tissue of rabbit inoculated with vaccinia virus. ^*∗*^Patients in these categories are not mutually exclusive. —, statistical analyses not performed.

**Table 2:** Prescription patterns according to gender and age.

			Initial daily dose			Maximum daily dose			Prescription period					
	*N* (%)		Mean (SD)			Mean (SD)			Mean (SD)			Median (25, 75 percentile)		
	Age													
	<65	≥65	<65a	≥65	*P* value	<65	≥65	*P* value	<65	≥65	*P* value	<65	≥65	*P* value
Sex														
Male	8578 (50.8)	13507 (47.5)	110.6 (57.8)	98.0 (58.4)	<0.001	149.7 (97.2)	130.4 (88.9)	<0.001	104.7 (118.4)	118.8 (126.5)	<0.001	49 (21,144)	57 (22,184)	<0.001
Female	8318 (49.2)	14928 (52.5)	101.9 (55.2)	86.6 (53.2)	<0.001	132.3 (85.3)	110.2 (75.7)	<0.001	100.5 (118.0)	116.2 (128.1)	<0.001	43 (16,132)	54 (21,178)	<0.001

Initial daily dose														
<50 mg	1470 (8.7)	4267 (15.0)	—	—	—	55.7 (55.6)	51.4 (54.7)	0.001	112.4 (124.5)	130.1 (132.2)	<0.001	56 (21,161)	63 (27,225)	<0.001
≥50 mg, <75 mg	2714 (16.1)	5977 (21.0)	—	—	—	86.2 (66.7)	78.8 (54.0)	<0.001	113.5 (122.5)	126.9 (129.1)	<0.001	56 (24,164)	65 (28,205)	<0.001
≥75 mg, <150 mg	4886 (28.9)	8253 (29.0)	—	—	—	116.2 (71.6)	109.8 (67.4)	<0.001	98.4 (114.7)	117.3 (127.4)	<0.001	44 (20,127)	56 (21,182)	<0.001
≥150 mg	7826 (46.3)	9938 (34.9)	—	—	—	191.9 (86.9)	182.2 (76.4)	<0.001	99.7 (117.4)	106.5 (123.0)	<0.001	42 (14,135)	46 (16,149)	0.013

**Table 3:** Subgroup analysis of individual disorders.

	Spinal disorders		*P* value	Postherpetic neuropathy		*P* value	Diabetic neuropathy		*P* value
	Outpatient	Inpatient		Outpatient	Inpatient		Outpatient	Inpatient	
*N*	21144	2358		2435	360		1244	234	
Sex (male), *N* (%)	10219 (48.3)	1211 (51.4)	0.006	1142 (46.9)	168 (46.7)	0.935	745 (59.9)	146 (62.4)	0.473
Age, mean (SD)	67.3 (13.9)	70.0 (13.1)	<0.001	69.1 (13.2)	70.8 (13.0)	0.030	67.7 (11.7)	69.1 (11.6)	0.084
Initial daily dose (mg), mean (SD)	89.5 (54.1)	101.4 (56.8)	<0.001	108.0 (58.7)	104.8 (52.6)	0.321	96.9 (54.2)	99.1 (57.8)	0.570
Maximum daily dose (mg), mean (SD)	115.2 (76.4)	140.0 (92.9)	<0.001	146.5 (96.9)	164.5 (106.2)	0.002	129.9 (85.1)	139.1 (92.6)	0.135
Prescription period (day)									
Mean (SD)	123.6 (128.2)	95.7 (116.1)	<0.001	95.2 (116.5)	94.0 (112.8)	0.857	160.3 (143.6)	103.2 (124.1)	<.0.001
Median (25, 75 percentile)	62 (28, 196)	43 (15, 122)	<0.001	42 (14, 119)	43.5 (20, 111.5)	0.600	91 (30, 365)	43 (17, 129)	<0.001
Prescription period over 90 days, *N* (%)	8726 (41.3)	724 (30.7)	<0.001	747 (30.7)	105 (29.2)	0.562	634 (51.0)	74 (31.6)	<0.001
Coprescribed oral drugs, mean (SD)	4.0 (3.7)	5.5 (4.2)	<0.001	4.3 (3.7)	5.4 (4.0)	<0.001	7.0 (4.4)	7.4 (4.6)	0.232
1-2-line drugs, *N* (%)	2798 (13.2)	272 (11.5)	0.021	350 (14.4)	49 (13.6)	0.670	171 (13.7)	31 (13.2)	0.839
Opioids, *N* (%)	881 (4.2)	322 (13.7)	<0.001	95 (3.9)	33 (9.2)	<0.001	33 (2.7)	21 (9.0)	<0.001
NSAIDs, *N* (%)	8813 (41.7)	1119 (47.5)	<0.001	959 (39.4)	164 (45.6)	0.026	264 (21.2)	63 (26.9)	0.054

	Cancer-related pain		*P* value	Trigeminal neuralgia		*P* value	Entrapment neuropathy of the upper limb		*P* value
	Outpatient	Inpatient		Outpatient	Inpatient		Outpatient	Inpatient	

*N*	2172	1761		713	67		1251	78	
Sex (male), *N*(%)	1167 (53.7)	1089 (61.8)	<0.001	320 (44.9)	31 (46.3)	0.828	499 (39.9)	37 (47.4)	0.188
Age, mean (SD)	64.3 (12.2)	65.0 (12.6)	0.060	65.9 (13.7)	68.9 (12.8)	0.079	65.4 (14.2)	68.7 (12.7)	0.044
Initial daily dose (mg), mean (SD)	108.3 (54.2)	107.1 (58.8)	0.515	109.9 (60.2)	117.4 (69.8)	0.337	84.4 (52.7)	93.1 (60.7)	0.161
Maximum daily dose (mg), mean (SD)	150.0 (96.4)	165.1 (116.7)	<0.001	148.7 (97.3)	165.5 (93.2)	0.176	107.8 (74.3)	142.8 (124.8)	<0.001
Prescription period (day)									
Mean (SD)	118.3 (123.2)	72.3 (92.4)	<0.001	124.0 (135.2)	76.6 (100.3)	0.006	125.6 (128.4)	126.9 (137.0)	0.935
Median (25, 75 percentile)	63 (28, 177)	36 (14, 84)	<0.001	51 (14, 220)	39 (16, 88)	0.371	63 (28, 204)	54 (21, 228)	0.631
Prescription period over 90 days, *N*(%)	888 (40.9)	418 (23.7)	<0.001	287 (40.3)	15 (22.4)	0.005	520	(41.6)	31 (39.7)
Coprescribed oral drugs, mean (SD)	5.1 (3.4)	5.7 (3.5)	<0.001	4.1 (4.0)	6.4 (4.3)	<0.001	3.8 (3.9)	5.8 (4.2)	<0.001
1-2-line drugs, *N*(%)	93 (4.3)	84 (4.8)	0.463	82 (11.5)	7 (10.4)	0.796	201 (16.1)	11 (14.1)	0.646
Opioids, *N*(%)	1001 (46.1)	1127 (64.0)	<0.001	25 (3.5)	8 (11.9)	0.001	28 (2.2)	7 (9.0)	<0.001
NSAIDs, *N*(%)	1111 (51.2)	1035 (58.8)	<0.001	161 (22.6)	33 (49.3)	<0.001	363 (29.0)	34 (43.6)	0.007

1-2-line drugs: first- and second-line drugs of the Japanese guideline.

**Table 4:** Analgesic drugs used before and after the pregabalin discontinuation.

	Before	After
*N*	39249	39249
Analgesics (*N*, %)		
Represcribed pregabalin use	—	4009 (10.2)

1-2-line drugs	5416 (13.8)	3213 (8.2)
Continued use	—	1761 (4.5)
New use	—	1348 (3.4)
Changed/added drugs	—	104 (0.3)
TCAs	837 (2.1)	448 (1.1)
Gabapentin	355 (0.9)	197 (0.5)
Neurotropin	3775 (9.6)	2106 (5.4)
Duloxetine	501 (1.3)	455 (1.2)
Mexiletine	310 (0.8)	221 (0.6)

Opioids	8171 (20.8)	5954 (15.2)
Continued use	—	1580 (4.0)
New use	—	2894 (7.4)
Changed/additions of drugs/route	—	1480 (3.8)

Oral opioids	5008 (12.8)	3350 (8.5)
Continued use	—	1358 (3.5)
New use	—	1649 (4.2)
Changed/added drugs	—	160 (0.4)
Changed/additions of route	—	183 (0.5)

Nonoral opioids	4737 (12.1)	3368 (8.6)
Continued use	—	702 (1.8)
New use	—	1465 (3.7)
Changed/added drugs	—	527 (1.3)
Changed/additions of route	—	674 (1.7)

Weak opioids	3238 (8.2)	2377 (6.1)
Continued use	—	710 (1.8)
New use	—	1444 (3.7)
Changed/added drugs	—	76 (0.2)
Changed/additions of the strength	—	147 (0.4)

Strong opioids	5928 (15.1)	3963 (10.1)
Continued use	—	1010 (2.6)
New use	—	1644 (4.2)
Changed/added drugs	—	1062 (2.7)
Changed/additions of the strength	—	247 (0.6)

1-2-line drugs:, first- and second-line drugs of the Japanese guideline; TCAs: tricyclic antidepressants (amitriptyline hydrochloride, imipramine hydrochloride, and nortriptyline hydrochloride); neurotropin: extract of cutaneous tissue of rabbit inoculated with vaccinia virus.

## Supplementary Materials

Supplementary Materials Table S1: subgroup analysis of characteristics of 12-month continuous pregabalin-prescribed patients. Table S2: subgroup analysis of individual disorders for analgesics drug use before and after the discontinuation of pregabalin.
