# Supplementary material for: Pregabalin Prescription for Neuropathic Pain and Fibromyalgia: A Descriptive Study Using Administrative Database in Japan
Source: Pain Res Manag. 2018 Jun 5;2018:2786151. doi: 10.1155/2018/2786151 (PMC6008692; doi:10.1155/2018/2786151)
Supplement: Supplementary Materials — Table S1: subgroup analysis of characteristics of 12-month continuous pregabalin-prescribed patients. Table S2: subgroup analysis of individual disorders for analgesics drug use before and after the discontinuation of pregabalin. [file 2786151.f1.docx]

Table S1. Subgroup analysis of characteristics of 12-month continuous pregabalin-prescribed patients.

|  | **All** | | **Outpatient** | | **Inpatient** | | ***P* value** |
| --- | --- | --- | --- | --- | --- | --- | --- |
| **N** | 6082 |  | 5396 |  | 686 |  |  |
| **Sex (Male) (N, %)** | 2942 | (48.4) | 2580 | (47.8) | 362 | (52.8) | 0.014 |
| **Age (Mean, SD)** | 68.9 | (11.9) | 68.9 | (11.9) | 68.4 | (11.8) | 0.315 |
| **Initial daily dose** |  |  |  |  |  |  |  |
| **(mg)(Mean, SD)** | 91.8 | (55.9) | 90.1 | (55.7) | 105.1 | (56.2) | <0.001 |
| **(N, %)** |  |  |  |  |  |  |  |
| **<50 mg** | 934 | (15.4) | 874 | (16.2) | 60 | (8.7) | - |
| **≥50 mg, <75 mg** | 1305 | (21.5) | 1200 | (22.2) | 105 | (15.3) | - |
| **≥75 mg, <150 mg** | 1718 | (28.2) | 1506 | (27.9) | 212 | (30.9) | - |
| **≥150 mg** | 2125 | (34.9) | 1816 | (33.7) | 309 | (45.0) | - |
| **Maximum daily dose** |  |  |  |  |  |  |  |
| **(mg)(Mean, SD)** | 153.5 | (106.2) | 148.8 | (102.0) | 190.3 | (129.3) | <0.001 |
| **(N, %)** |  |  |  |  |  |  |  |
| **<150 mg** | 2347 | (38.6) | 2186 | (40.5) | 161 | (23.5) | - |
| **≥150 mg, <300 mg** | 2740 | (45.1) | 2374 | (44.0) | 366 | (53.4) | - |
| **≥300 mg** | 995 | (16.4) | 836 | (15.5) | 159 | (23.2) | - |
| **Change in dose (N, %)** | 3143 | (51.7) | 2739 | (50.8) | 404 | (58.9) | <0.001 |
| **Co-prescribed oral drugs (Mean, SD)** | 5.2 | (3.9) | 5.1 | (3.9) | 6.0 | (3.9) | <0.001 |
| **Co-prescribed analgesics (N, %)** |  |  |  |  |  |  |  |
| **1-2-line drugs** | 821 | (13.5) | 746 | (13.8) | 75 | (10.9) | 0.037 |
| **TCAs** | 124 | (2.0) | 110 | (2.0) | 14 | (2.0) | - |
| **Gabapentin** | 42 | (0.7) | 34 | (0.6) | 8 | (1.2) | - |
| **Neurotropin®** | 609 | (10.0) | 569 | (10.5) | 40 | (5.8) | - |
| **Duloxetine** | 52 | (0.9) | 44 | (0.8) | 8 | (1.2) | - |
| **Mexiletine** | 50 | (0.8) | 41 | (0.8) | 9 | (1.3) | - |
| **Opioids** | 389 | (6.4) | 303 | (5.6) | 86 | (12.5) | <0.001 |
| **Oral opioids** | 327 | (5.4) | 255 | (4.7) | 72 | (10.5) | - |
| **Non-oral opioids** | 59 | (1.0) | 45 | (0.8) | 14 | (2.0) | - |
| **Strong opioids** | 156 | (2.6) | 113 | (2.1) | 43 | (6.3) | - |
| **Weak opioid** | 239 | (3.9) | 194 | (3.6) | 45 | (6.6) | - |
| **NSAIDs** | 2233 | (36.7) | 1966 | (36.4) | 267 | (38.9) | 0.203 |
| **Neuropathic pain-related disorders^*^ (N, %)** |  |  |  |  |  |  |  |
| **Spinal disorders** | 3525 | (58.0) | 3267 | (60.5) | 258 | (37.6) | <0.001 |
| **Post herpetic neuropathy** | 294 | (4.8) | 257 | (4.8) | 37 | (5.4) | 0.468 |
| **Diabetic neuropathy** | 349 | (5.7) | 317 | (5.9) | 32 | (4.7) | 0.199 |
| **Cancer-related pain** | 356 | (5.9) | 270 | (5.0) | 86 | (12.5) | <0.001 |
| **Trigeminal neuralgia** | 127 | (2.1) | 122 | (2.3) | 5 | (0.7) | 0.008 |
| **Entrapment peripheral neuropathy of the upper limb** | 212 | (3.5) | 196 | (3.6) | 16 | (2.3) | 0.080 |
| **Other neuropathic pain** | 1504 | (24.7) | 1313 | (24.3) | 191 | (27.8) | - |
| **Fibromyalgia** | 27 | (0.4) | 24 | (0.4) | 3 | (0.4) | - |
| **Other** | 277 | (4.6) | 160 | (3.0) | 117 | (17.1) | - |

1-2-line drugs, first and second-line drugs of the Japanese guideline; TCAs, tricyclic antidepressants (amitriptyline hydrochloride, imipramine hydrochloride and nortriptyline hydrochloride); Neurotropin®, extract of cutaneous tissue of rabbit inoculated with vaccinia virus.

*: Patients in these categories are not mutually exclusive.

– signifies statistical analyses not performed.

Table S2. Subgroup analysis of individual disorders for analgesics drug use before and after the discontinuation of pregabalin.

|  | **Spinal Disorders** | | | | **Post Herpetic Neuropathy** | | | | **Diabetic Neuropathy** | | | |
| --- | --- | --- | --- | --- | --- | --- | --- | --- | --- | --- | --- | --- |
|  | **Before** | | **After** | | **Before** | | **After** | | **Before** | | **After** | |
| **N** | 19977 | | 19977 | | 2501 | | 2501 | | 1129 | | 1129 | |
| **Analgesics (N, %)** |  |  |  |  |  |  |  |  |  |  |  |  |
| **Re-pregabalin use** |  |  | 2290 | (11.5) |  |  | 196 | (7.8) |  |  | 168 | (14.9) |
| **1-2-line drugs** | 3213 | (16.1) | 1921 | (9.6) | 527 | (21.1) | 262 | (10.5) | 232 | (20.5) | 174 | (15.4) |
| **Continued use** |  |  | 1160 | (5.8) |  |  | 153 | (6.1) |  |  | 85 | (7.5) |
| **New use** |  |  | 707 | (3.5) |  |  | 96 | (3.8) |  |  | 77 | (6.8) |
| **Changed/added drugs** |  |  | 54 | (0.3) |  |  | 13 | (0.5) |  |  | 12 | (1.1) |
| **TCAs** | 283 | (1.4) | 191 | (1.0) | 200 | (8.0) | 75 | (3.0) | 27 | (2.4) | 19 | (1.7) |
| **Gabapentin** | 99 | (0.5) | 69 | (0.3) | 65 | (2.6) | 29 | (1.2) | 15 | (1.3) | 13 | (1.2) |
| **Neurotropin®** | 2727 | (13.7) | 1531 | (7.7) | 280 | (11.2) | 144 | (5.8) | 85 | (7.5) | 64 | (5.7) |
| **Duloxetine** | 142 | (0.7) | 144 | (0.7) | 35 | (1.4) | 28 | (1.1) | 48 | (4.3) | 44 | (3.9) |
| **Mexiletine** | 120 | (0.6) | 98 | (0.5) | 30 | (1.2) | 23 | (0.9) | 82 | (7.3) | 54 | (4.8) |
| **Opioids** | 3040 | (15.2) | 2632 | (13.2) | 267 | (10.7) | 196 | (7.8) | 120 | (10.6) | 123 | (10.9) |
| **Continued use** |  |  | 569 | (2.8) |  |  | 64 | (2.6) |  |  | 30 | (2.7) |
| **New use** |  |  | 1680 | (8.4) |  |  | 100 | (4.0) |  |  | 78 | (6.9) |
| **Changed/additions of drugs/route** |  |  | 383 | (1.9) |  |  | 32 | (1.3) |  |  | 15 | (1.3) |
| **Oral-opioids** | 1789 | (9.0) | 1618 | (8.1) | 193 | (7.7) | 130 | (5.2) | 74 | (6.6) | 76 | (6.7) |
| **Continued use** |  |  | 522 | (2.6) |  |  | 58 | (2.3) |  |  | 25 | (2.2) |
| **New use** |  |  | 974 | (4.9) |  |  | 61 | (2.4) |  |  | 47 | (4.2) |
| **Changed/added drugs** |  |  | 45 | (0.2) |  |  | 4 | (0.2) |  |  | 2 | (0.2) |
| **Changed/additions of route** |  |  | 77 | (0.4) |  |  | 7 | (0.3) |  |  | 2 | (0.2) |
| **Non-oral opioids** | 1563 | (7.8) | 1238 | (6.2) | 108 | (4.3) | 90 | (3.6) | 70 | (6.2) | 53 | (4.7) |
| **Continued use** |  |  | 151 | (0.8) |  |  | 18 | (0.7) |  |  | 8 | (0.7) |
| **New use** |  |  | 811 | (4.1) |  |  | 49 | (2.0) |  |  | 35 | (3.1) |
| **Changed/added drugs** |  |  | 91 | (0.5) |  |  | 11 | (0.4) |  |  | 4 | (0.4) |
| **Changed/additions of route** |  |  | 185 | (0.9) |  |  | 12 | (0.5) |  |  | 6 | (0.5) |
| **Weak opioids** | 1626 | (8.1) | 1484 | (7.4) | 145 | (5.8) | 94 | (3.8) | 63 | (5.6) | 68 | (6.0) |
| **Continued use** |  |  | 425 | (2.1) |  |  | 38 | (1.5) |  |  | 20 | (1.8) |
| **New use** |  |  | 952 | (4.8) |  |  | 49 | (2.0) |  |  | 44 | (3.9) |
| **Changed/added drugs** |  |  | 34 | (0.2) |  |  | 1 | (0.0) |  |  | 2 | (0.2) |
| **Changed/additions of the strength** |  |  | 73 | (0.4) |  |  | 6 | (0.2) |  |  | 2 | (0.2) |
| **Strong opioids** | 1712 | (8.6) | 1343 | (6.7) | 145 | (5.8) | 112 | (4.5) | 78 | (6.9) | 62 | (5.5) |
| **Continued use** |  |  | 201 | (1.0) |  |  | 33 | (1.3) |  |  | 12 | (1.1) |
| **New use** |  |  | 850 | (4.3) |  |  | 54 | (2.2) |  |  | 39 | (3.5) |
| **Changed/added drugs** |  |  | 166 | (0.8) |  |  | 20 | (0.8) |  |  | 7 | (0.6) |
| **Changed/additions of the strength** |  |  | 126 | (0.6) |  |  | 5 | (0.2) |  |  | 4 | (0.4) |

1-2-line drugs, first and second-line drugs of the Japanese guideline; TCAs, tricyclic antidepressants (amitriptyline hydrochloride, imipramine hydrochloride and nortriptyline hydrochloride); Neurotropin®, extract of cutaneous tissue of rabbit inoculated with vaccinia virus.

Table S2. (Continued)

|  | **Cancer-Related Pain** | | | | **Trigeminal Neuralgia** | | | | **Entrapment Neuropathy of the Upper Limb** | | | |
| --- | --- | --- | --- | --- | --- | --- | --- | --- | --- | --- | --- | --- |
|  | **Before** | | **After** | | **Before** | | **After** | | **Before** | | **After** | |
| **N** | 3577 | | 3577 | | 653 | | 653 | | 1117 | | 1117 | |
| **Analgesics (N, %)** |  |  |  |  |  |  |  |  |  |  |  |  |
| **Re-pregabalin use** |  |  | 290 | (8.1) |  |  | 87 | (13.3) |  |  | 127 | (11.4) |
| **1-2-line drugs** | 419 | (11.7) | 217 | (6.1) | 114 | (17.5) | 82 | (12.6) | 203 | (18.2) | 128 | (11.5) |
| **Continued use** |  |  | 91 | (2.5) |  |  | 50 | (7.7) |  |  | 74 | (6.6) |
| **New use** |  |  | 110 | (3.1) |  |  | 30 | (4.6) |  |  | 52 | (4.7) |
| **Changed/added drugs** |  |  | 16 | (0.4) |  |  | 2 | (0.3) |  |  | 2 | (0.2) |
| **TCAs** | 98 | (2.7) | 39 | (1.1) | 31 | (4.7) | 25 | (3.8) | 12 | (1.1) | 12 | (1.1) |
| **Gabapentin** | 121 | (3.4) | 52 | (1.5) | 21 | (3.2) | 14 | (2.1) | 5 | (0.4) | 3 | (0.3) |
| **Neurotropin®** | 85 | (2.4) | 40 | (1.1) | 48 | (7.4) | 29 | (4.4) | 185 | (16.6) | 106 | (9.5) |
| **Duloxetine** | 109 | (3.0) | 84 | (2.3) | 14 | (2.1) | 10 | (1.5) | 9 | (0.8) | 7 | (0.6) |
| **Mexiletine** | 54 | (1.5) | 24 | (0.7) | 11 | (1.7) | 11 | (1.7) | 7 | (0.6) | 8 | (0.7) |
| **Opioids** | 2716 | (75.9) | 1797 | (50.2) | 62 | (9.5) | 71 | (10.9) | 95 | (8.5) | 117 | (10.5) |
| **Continued use** |  |  | 752 | (21.0) |  |  | 20 | (3.1) |  |  | 16 | (1.4) |
| **New use** |  |  | 194 | (5.4) |  |  | 39 | (6.0) |  |  | 92 | (8.2) |
| **Changed/additions of drugs/route** |  |  | 851 | (23.8) |  |  | 12 | (1.8) |  |  | 9 | (0.8) |
| **Oral-opioids** | 2269 | (63.4) | 892 | (24.9) | 38 | (5.8) | 41 | (6.3) | 45 | (4.0) | 65 | (5.8) |
| **Continued use** |  |  | 649 | (18.1) |  |  | 16 | (2.5) |  |  | 10 | (0.9) |
| **New use** |  |  | 121 | (3.4) |  |  | 21 | (3.2) |  |  | 51 | (4.6) |
| **Changed/added drugs** |  |  | 91 | (2.5) |  |  | 2 | (0.3) |  |  | 0 | (0.0) |
| **Changed/additions of route** |  |  | 31 | (0.9) |  |  | 2 | (0.3) |  |  | 4 | (0.4) |
| **Non-oral opioids** | 1482 | (41.4) | 1301 | (36.4) | 35 | (5.4) | 35 | (5.4) | 61 | (5.5) | 56 | (5.0) |
| **Continued use** |  |  | 430 | (12.0) |  |  | 7 | (1.1) |  |  | 7 | (0.6) |
| **New use** |  |  | 114 | (3.2) |  |  | 20 | (3.1) |  |  | 44 | (3.9) |
| **Changed/added drugs** |  |  | 335 | (9.4) |  |  | 5 | (0.8) |  |  | 3 | (0.3) |
| **Changed/additions of route** |  |  | 422 | (11.8) |  |  | 3 | (0.5) |  |  | 2 | (0.2) |
| **Weak opioids** | 671 | (18.8) | 204 | (5.7) | 23 | (3.5) | 28 | (4.3) | 43 | (3.8) | 66 | (5.9) |
| **Continued use** |  |  | 112 | (3.1) |  |  | 8 | (1.2) |  |  | 10 | (0.9) |
| **New use** |  |  | 50 | (1.4) |  |  | 19 | (2.9) |  |  | 52 | (4.7) |
| **Changed/added drugs** |  |  | 15 | (0.4) |  |  | 0 | (0.0) |  |  | 1 | (0.1) |
| **Changed/additions of the strength** |  |  | 27 | (0.8) |  |  | 1 | (0.2) |  |  | 3 | (0.3) |
| **Strong opioids** | 2462 | (68.8) | 1678 | (46.9) | 45 | (6.9) | 44 | (6.7) | 61 | (5.5) | 56 | (5.0) |
| **Continued use** |  |  | 687 | (19.2) |  |  | 12 | (1.8) |  |  | 7 | (0.6) |
| **New use** |  |  | 164 | (4.6) |  |  | 21 | (3.2) |  |  | 44 | (3.9) |
| **Changed/added drugs** |  |  | 765 | (21.4) |  |  | 9 | (1.4) |  |  | 3 | (0.3) |
| **Changed/additions of the strength** |  |  | 62 | (1.7) |  |  | 2 | (0.3) |  |  | 2 | (0.2) |

1-2-line drugs, first and second-line drugs of the Japanese guideline; TCAs, tricyclic antidepressants (amitriptyline hydrochloride, imipramine hydrochloride and nortriptyline hydrochloride); Neurotropin®, extract of cutaneous tissue of rabbit inoculated with vaccinia virus.
